# Association between rs2853669 in TERT gene and the risk and prognosis of human cancer: a systematic review and meta-analysis

**DOI:** 10.18632/oncotarget.15140

**Published:** 2017-02-07

**Authors:** Na Shen, Yanjun Lu, Xiong Wang, Jing Peng, Yaowu Zhu, Liming Cheng

**Affiliations:** ^1^ Department of Laboratory Medicine, Tongji Hospital, Tongji Medical College, Huazhong University of Science and Technology, Wuhan 430030, China

**Keywords:** *TERTp* mutations, rs2853669, risk, prognosis, meta-analysis

## Abstract

The polymorphism rs2853669 within the promoter of telomerase reverse transcriptase gene (*TERTp*) has been debated about its role in cancer risk and prognosis. Additionally, several studies report inconsistent results concerning the modifying effect of rs2853669 on the prognostic value of *TERTp* mutations in cancer patients. Here, we performed this meta-analysis to comprehensively evaluate the role of rs2853669 in the risk and prognosis of human cancer, and further assess its modifying impact on *TERTp* mutations in the survival of cancer patients. We systematically searched literature via PubMed, Web of Science, and EMBASE through July 2016, and included 22 eligible studies. The overall analysis (64,119 cases and 78,988 controls) demonstrated that rs2853669 did not increase or decrease the overall cancer risk. Subsequent analyses also did not reveal any association between rs2853669 and overall cancer prognosis. However, we identified a modifying effect of rs2853669 on *TERTp* mutations in that, among cancer patients with *TERTp* mutations, only those carrying the TT genotype had a poor survival (Hazard ratio = 1.56, 95% confidence interval = 1.06-2.28); subgroup analyses by cancer type also supported these results. In conclusion, our findings suggest that rs2853669 could be important for assessing the prognostic value of *TERTp* mutations. Future large studies are required to further validate our results.

## INTRODUCTION

Human cancer is a major global health problem. There were approximately 14 million new cancer cases in 2012 worldwide, and by 2025, the predicted global cancer incidence will rise to 20 million [1]. Cancer is the leading cause of death in China [2] and the second leading cause of death in the United States [3]. Although environmental risk factors are important in cancer pathogenesis, genetic predisposition also has a confirmed crucial role in the risk and prognosis of cancer. Notably, genetic alterations in the proximal promoter of the telomerase reverse transcriptase gene (*TERT*) are significantly associated with many cancer types [4].

The *TERT* gene encodes the key catalytic subunit of telomerase, which is important for the maintenance of chromosome stability [5]. Deregulation of *TERT* often results in abnormal telomerase activation and causes unlimited cell proliferation and even malignancies [4]. An increasing number of studies suggest that somatic mutations (e.g., -124C>T and -146C>T) and single nucleotide polymorphisms (SNPs) within the *TERT* promoter (*TERTp*) could influence the susceptibility and prognosis of human cancers [6–12]. In particular, the SNP rs2853669 (T > C), located at -245 bp from the *TERT* ATG site, is associated with the risk of various cancers such as breast cancer [6], lung cancer [13], and hepatocellular carcinoma [14]; however, these results remain inconclusive. Some studies also supported a prognostic effect of rs2853669 on cancer prognosis [15, 16] whereas other studies have disproved this conclusion [17, 18]. This SNP may have a modifying role in the prognostic value of *TERTp* mutations. For example, Ko et al. [11] showed that C carriers of rs2853669 in conjunction with *TERTp* mutations had a poor prognosis in liver cancer. By contrast, Nagore et al. [16] reported a poor melanoma survival only in cancer patients with both *TERTp* mutations and the TT genotype.

These conflicting results may be because of the insufficient power of individual studies. Therefore, we comprehensively searched the relevant literature and performed a meta-analysis to systematically evaluate the individual effect of rs2853669 on cancer risk and prognosis, and further assess its modifying impact on *TERTp* mutations with regard to the survival of cancer patients.

## RESULTS

### Literature search

**Figure 1:**
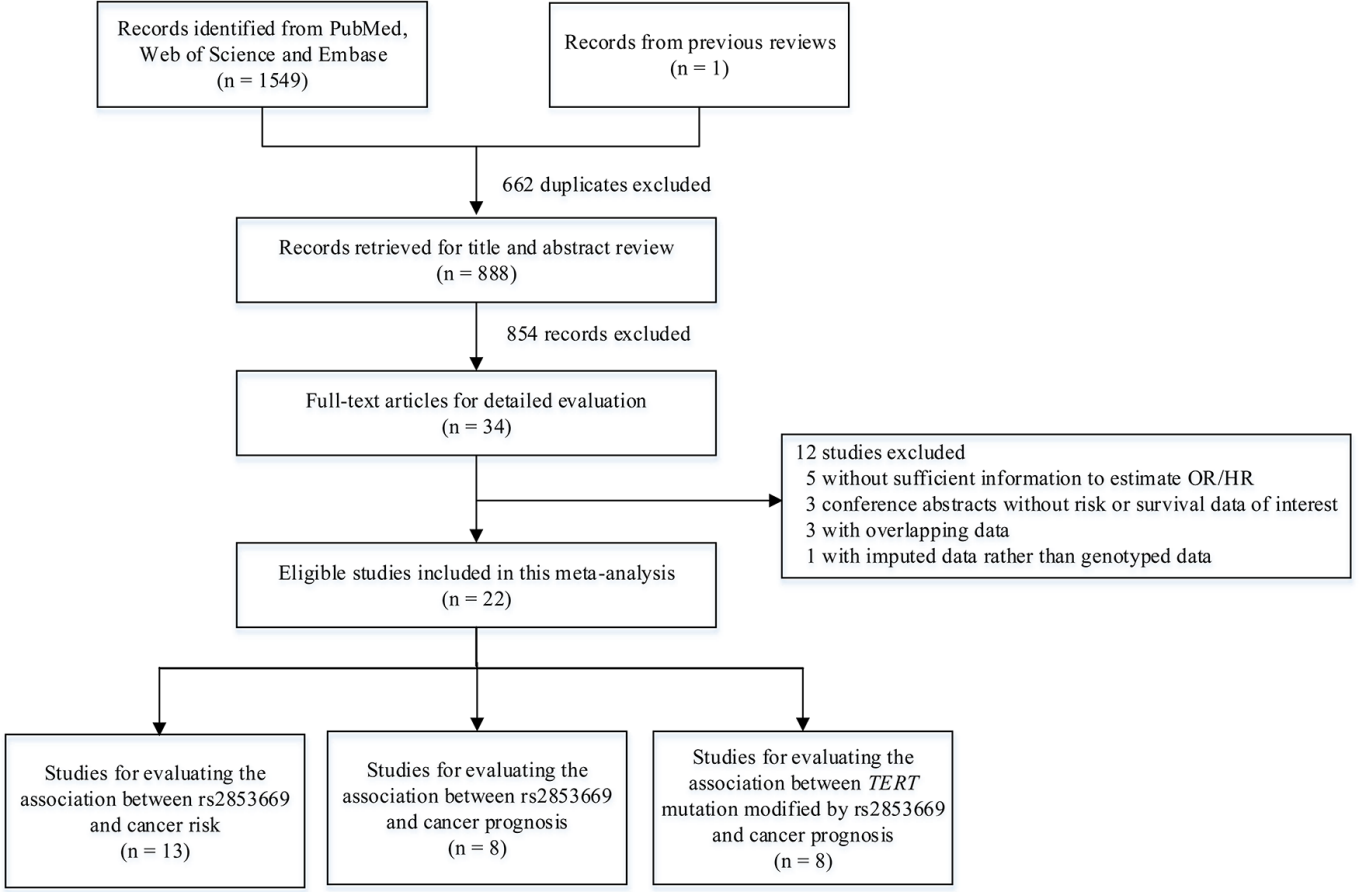
A flow chart of literature search

As shown in Figure 1, 1,550 records were comprehensively identified by the literature search. After removing 662 duplicate records and 866 irrelevant records, we finally included 22 eligible studies in this meta-analysis [6–8, 11–29]. Of these studies, 13 studies focused on the association between rs2853669 and cancer risk [6, 7, 12–14, 19, 23–29], eight studies focused on the association between rs2853669 and cancer prognosis [15–18, 20, 21, 24, 25], and eight studies focused on the modifying effect of rs2853669 on *TERTp* mutations [8, 11, 15–18, 21, 22].

### Association between rs2853669 and cancer risk

Thirteen eligible studies involving 16 datasets were pooled to evaluate the association between rs2853669 and cancer risk; these studies comprised 64,119 patients and 78,988 controls [6, 7, 12–14, 19, 23–29]. As shown in Table 1, four studies focused on breast cancer [6, 12, 19, 29], two studies focused on lung cancer [13, 27], one study focused on breast cancer and ovarian cancer [7], one study focused on prostate cancer and breast cancer [26], and the remaining studies each focused on a different cancer type [14, 23–25, 28]. Nine studies were conducted in Caucasians [6, 12, 19, 23–26, 28, 29], three studies in Asians [13, 14, 27], and one study in a mixed-ethnic population [7]. Most studies were of high quality (i.e., scores >9), except for three studies (i.e., scores ≤9) [7, 14, 23]. In the overall analyses, rs2853669 was not significantly associated with cancer risk in the allelic, dominant, recessive, codominant, or additive model (Table 2). Because of significant heterogeneity among the studies, we performed a meta-regression analysis to explore the source of heterogeneity by considering as potential covariates the publication year, cancer type, ethnicity, sample size (>1000 vs. ≤1000) and quality scores (>9 vs. ≤9). The results did not identify any significant covariate contributing to heterogeneity in all genetic models (data not shown). We further conducted subgroup analyses by cancer type (Supplementary Figure 1a), and observed that the rs2853669 C allele seemed to be protective against breast cancer in the allelic model (odds ratio (OR) = 0.95, 95% confidence interval (CI) = 0.93-0.97, *P_heterogeneity_* = 0.401, *I^2^* = 3.7%). Further one-way sensitivity analyses showed that our results were stable (Supplementary Figure 2) and the Begg's test and Egger's test revealed no publication bias in any genetic model (Table 2 and Supplementary Figure 3).

**Table 1:** Characteristics of studies included for the association between rs2853669 and cancer risk

First author	Publicationyear	Country	Cancertype	Ethnicity	Sex	Cases	Controls	Scores
Savage [6]	2007	Poland	BC	Caucasian	F	1,995	2,296	14
Varadi [19]	2009	Poland	BC	Caucasian	F	841	460	13
Varadi [19]	2009	Sweden	BC	Caucasian	F	815	1,559	12
Shen [12]	2010	USA	BC	Caucasian	F	1,067	1,110	13
Park [14]	2010	Korea	HCC	Asian	NA	290	277	2
Bojesen [7]	2013	Mixed	BC	Mixed	F	46,451	45,299	5
Bojesen [7]	2013	Mixed	OC	Mixed	F	9,357	23,491	5
Zhong [13]	2013	China	LC	Asian	F/M	502	502	14
Jannuzzi [23]	2015	Turkey	CRC	Caucasian	F/M	104	135	9
Mosrati [25]	2015	Sweden	AML	Caucasian	F/M	226	779	12
Mosrati [24]	2015	Sweden	GBM	Caucasian	F/M	128	779	10
Shadrina [26]	2015	Russia	PC	Caucasian	M	372	363	10
Shadrina [26]	2015	Russia	BC	Caucasian	F	660	523	11
Yoo [27]	2015	Korea	LC	Asian	F/M	1,100	1,096	13
Bayram [28]	2016	Turkey	GC	Caucasian	F/M	104	209	11
Oztas [29]	2016	Turkey	BC	Caucasian	F	107	110	10

BC, breast cancer; HCC, hepatocarcinoma; OC, ovarian cancer; LC, lung cancer; CRC, colorectal cancer; AML, acute myeloid leukemia; GBM, glioblastoma; PC, prostate cancer; GC, gastric cancer; F, female; M, male; NA, not available.

**Table 2:** Meta-analyses for the individual effect of rs2853669 on the risk and overall survival of cancer

Genetic model^a^	Statistics			Heterogeneity		Publication bias	
	N	Effectsize^b^	95%CI	*P_heterogeneity_*	*I^2^* (%)	*P_Begg_*	*P_Egger_*
**Assessment of cancer risk**							
Allelic model	18	1.01	0.96-1.06	< 0.001	77.5	0.880	0.142
Dominant model	14	1.06	0.94-1.20	< 0.001	65.6	1.000	0.832
Recessive model	14	1.00	0.81-1.23	< 0.001	78.0	0.443	0.663
Codominant model (TC vs. TT)	14	1.07	0.97-1.19	0.030	46.1	0.743	0.621
Codominant model (CC vs. TT)	14	1.03	0.80-1.34	< 0.001	80.3	0.584	0.986
Additive model	14	1.03	0.93-1.15	< 0.001	77.2	0.827	0.860
**Assessment of cancer overall survival**							
Dominant model	6	0.87	0.63-1.20	0.021	62.2	0.452	0.264
Codominant model (TC vs. TT)	3	1.11	0.86-1.43	0.318	12.6	1.000	0.241
Codominant model (CC vs. TT)	3	1.80	1.09-2.97	0.105	55.7	1.000	0.784

^a^ Allelic model refers to C allele versus T allele; Dominant model refers to TC+CC versus TT; Recessive model refers to CC versus TC+TT.

^b^ Effect size in the assessment of cancer risk refers to OR, and effect size in the assessment of cancer overall survival refers to HR.

### Association between rs2853669 and cancer prognosis

There were eight eligible studies, which included 2,234 cases, used to evaluate the association between rs2853669 and cancer prognosis (Table 3) [15–18, 20, 21, 24, 25]. Of these, five studies focused on glioblastoma [15, 17, 18, 21, 24], and the remaining studies focused on a separate cancer type [16, 20, 25]. Two genetic models, including dominant and codominant models, were used in these studies; therefore, we pooled the survival data, based on the two genetic models. The results were that rs2853669 was not associated with the overall survival (OS) of cancer in the dominant or TC vs. TT codominant model (Table 2 and Supplementary Figure 4a and 4b), and the sensitivities in these models exhibited robust results (Supplementary Figure 5a and 5b). There seemed to be a significant association in the CC versus TT codominant model between rs2853669 and cancer OS (hazards ratio (HR) = 1.80, 95%CI = 1.09-2.97, *P_heterogeneity_* = 0.105, *I^2^* = 55.7%); however, the small number of studies (n = 3) limited its credibility (Table 2 and Supplementary Figure 4c). Moreover, further sensitivity analysis suggested that the result was unstable when eliminating the studies of Mosrati et al. on glioblastoma [24] (HR = 1.46, 95%CI = 0.96-2.21, *P_heterogeneity_* = 0.304, *I^2^* = 5.5%) or acute myeloid leukemia (HR = 1.82, 95%CI=0.72-4.52, *P_heterogeneit_*_y_ = 0.034, *I^2^* = 77.6%) (Supplementary Figure 5c) [25]. We then performed subgroup analyses by cancer type in the two genetic models, and found that rs2853669 also had no association with the OS of glioblastoma (Supplementary Figure 4). In addition, no publication bias was identified in these two genetic models (Table 2 and Supplementary Figure 6).

**Table 3:** Characteristics of studies included for the individual and modifying effect of rs2853669 on cancer prognosis

First author	Publicationyear	Country	Cancertype	Sex	Follow-up	Cases	Outcomes
**Individual effect of rs2853669**							
Shen [20]	2012	USA	BC	F	The mean follow-up time was 8.0 years (range: 0.2–9.4 years).	1,102	BCSM
Park [21]	2014	Korea	GBM	F/M	> 1500 days for OS and > 800 days for PFS	48	OS; PFS
Mosrati [24]	2015	Sweden	GBM	F/M	> 60 months	92	OS
Mosrati [25]	2015	Sweden	AML	F/M	> 120 months	226	OS
Simon [17]	2015	Germany	GBM	F/M	The mean follow-up was 16.5+15.3 months (median: 12.0; range: 1–97)	176	OS
Spiegl-Kreinecker [15]	2015	Austria	GBM	F/M	> 150 months	126	OS
Batista [18]	2016	Portugal and Brazil	GBM	F/M	> 125 months	164	OS
Nagore [16]	2016	Spain	LICM	F/M	The median follow-up was 47 months (95% CI 39–56).	300	MSS; DFS
**Modifying effect of rs2853669 on *TERTp* mutations**							
Rachakonda [8]	2013	Sweden	BLC	F/M	15 years	327	OS; RFS
Park [21]	2014	Korea	GBM	F/M	> 1500 days for OS and > 800 days for PFS	48	OS; PFS
Hosen [22]	2015	Germany and Sweden	CCRCC	F/M	NA	188	DFS
Simon [17]	2015	Germany	GBM	F/M	The mean follow-up was 16.5+15.3 months (median: 12.0; range: 1-97)	176	OS
Spiegl-Kreinecker [15]	2015	Austria	GBM	F/M	> 150 months	67	OS
Batista [18]	2016	Portugal and Brazil	GBM	F/M	> 125 months	504	OS
Ko [11]	2016	Korea	HCC	F/M	> 60 months	165	OS; RFS
Nagore [16]	2016	Spain	LICM	F/M	The median follow-up was 47 months (95% CI 39-56).	300	OS; DFS

BC, breast cancer; AML, acute myeloid leukemia; GBM, glioblastoma; LICM, localized invasive cutaneous melanoma; BLC, bladder cancer; CCRCC, clear cell renal cell carcinoma; HCC, hepatocellular carcinoma; F, female; M, male; NA, not available; BSCM, breast cancer-specific mortality; OS, overall survival; PFS, progression-free survival; MSS, melanoma-specific survival; DFS, disease-free survival; RFS, recurrence-free survival.

Because only two studies assessing the prognostic effect of rs2853669 on cancer-free survival were identified in the literature, we failed to conduct a meta-analysis on this issue. However, these two studies did not find an association of rs2853669 with progression-free survival (PFS) [21] or with disease-free survival (DFS) [16] in cancer patients.

### The modifying effect of rs2853669 on *TERTp* mutations

**Figure 2:**
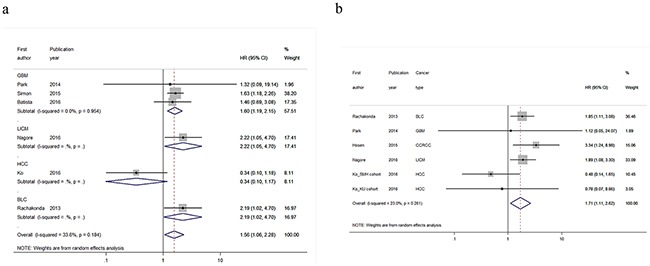
Forest plots of the modifying effect of rs2853669 on *TERTp* mutations for **a.** overall survival and **b.** disease-free survival in cancer patients carrying TT genotype. Datasets/studies that failed to provide relevant HRs were excluded from the forest plots. GBM, glioblastoma; LICM, localized invasive cutaneous melanoma; HCC, hepatocellular carcinoma; BLC, bladder cancer; CCRCC, clear cell renal cell carcinoma.

**Figure 3:**
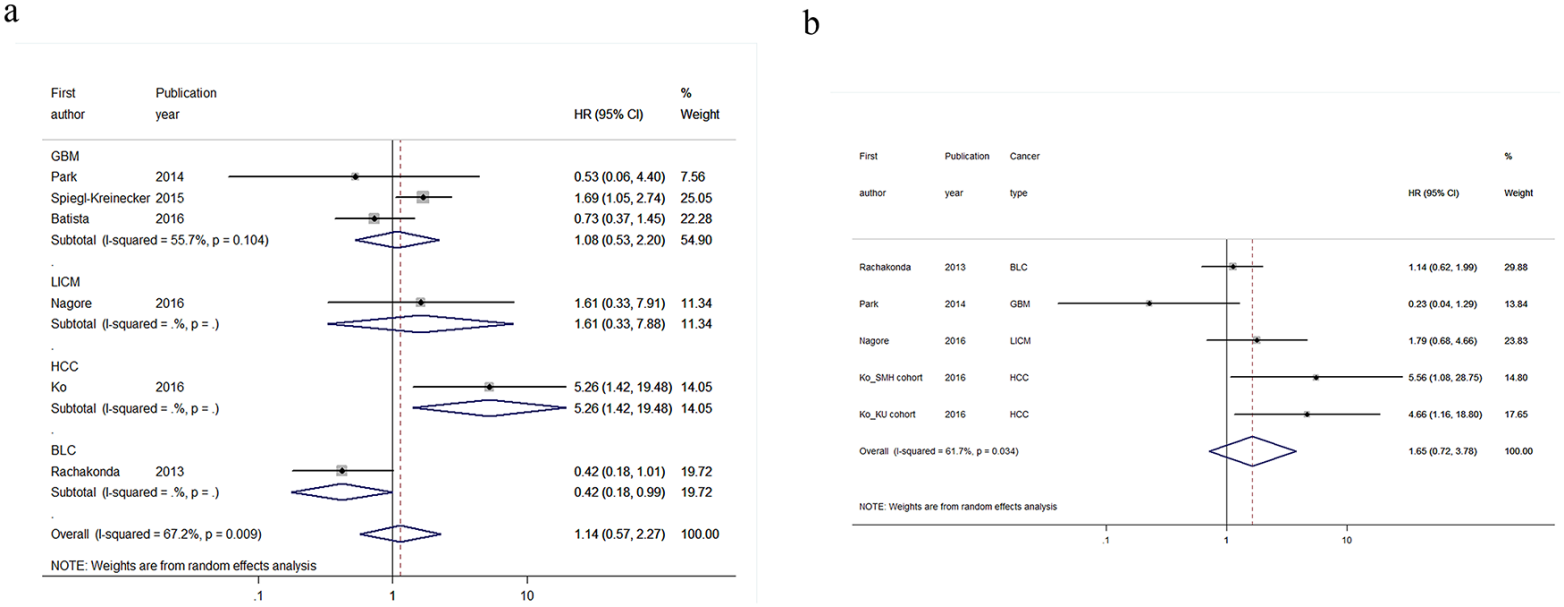
Forest plots of the modifying effect of rs2853669 on *TERTp* mutations for **a.** overall survival and **b.** disease-free survival in cancer patients carrying TC or CC genotype. Datasets/studies that failed to provide relevant HRs were excluded from the forest plots. GBM, glioblastoma; LICM, localized invasive cutaneous melanoma; HCC, hepatocellular carcinoma; BLC, bladder cancer; CCRCC, clear cell renal cell carcinoma.

Eight studies comprising 1,775 cases were included to evaluate the modifying effect of rs2853669 on the prognostic value of *TERTp* mutations for cancer survival (Table 3) [8, 11, 15–18, 21, 22]. Of these, four studies focused on glioblastoma [15, 17, 18, 21], and the other studies focused on bladder cancer [8], clear cell renal cell carcinoma [22], hepatocellular carcinoma [11], and localized invasive cutaneous melanoma [16]. Interestingly, we observed that *TERTp* mutations conferred worse OS only in cancer patients carrying the rs2853669 TT genotype (Figure 2a, HR = 1.56, 95%CI=1.06-2.28, *P_heterogeneit_*_y_ = 0.184, *I^2^* = 33.6%); this association was particularly evident in glioblastoma patients (Figure 2a, HR = 1.60, 95%CI=1.19-2.15, *P_heterogeneit_*_y_ = 0.954, *I^2^* = 0.0%). Moreover, a similar result was also demonstrated in the DFS of cancer patients (Figure 2b, HR = 1.71, 95%CI=1.11-2.62, *P_heterogeneit_*_y_ = 0.261, *I^2^* = 23.0%). In hepatocellular carcinoma, the rs2853669 seemed to play an opposite role than in other cancer type. But the only one relevant study [11] hindered us to perform subgroup analysis to explore the potential effect of rs2853669 on this cancer type. However, in cancer patients who were rs2853669 C carriers, we did not observe any significant result in the analysis of OS (Figure 3a) or DFS (Figure 3b), which suggested rs2853669 had an important role in determining the prognostic impact of *TERTp* mutations in human cancer. The Begg's test and Egger's test showed no publication bias for the aforementioned analyses (Supplementary Figure 7; OS for TT genotype: *P_Begg_*= 0.452, *P_Egger_* = 0.518; OS for C carriers: *P_Begg_*= 1, *P_Egger_* = 0.877; DFS for TT genotype: *P_Begg_*= 0.707, *P_Egger_* = 0.404; DFS for C carriers: *P_Begg_*= 0.1, *P_Egger_* = 0.660). We also performed sensitivity analyses and found that the pooled HR was not materially changed for cancer OS in rs2853669 C carriers (Supplementary Figure 8b), but the pooled HRs were not stable for cancer OS in rs2853669 TT genotypes (Supplementary Figure 8a) or for cancer DFS (Supplementary Figure 8c and 8d).

## DISCUSSION

The *TERT* gene is important to maintain chromosomal stability and mutations within its promoter region exist in several human cancers. Recently, inconsistent results were reported regarding the effect of rs2853669 in the *TERT* gene on the risk and prognosis of cancer [6–8, 11–29]. Thus we conducted this meta-analysis to comprehensively assess its individual role in cancer risk/prognosis and its modifying effect on *TERTp* mutations.

We observed that rs2853669 alone did not increase or decrease the risk and prognosis of cancer. However, when we performed subgroup analyses by cancer type, we interestingly found that rs2853669 may decrease breast cancer risk in the allelic model, which is in accordance with the findings of two published meta-analyses [30, 31]. A possible explanation for the contradictions between the overall results and subgroup results is that rs2853669 may have a tissue-specific effect. Several studies have shown its function on affecting *TERT* expression and telomerase activity [8, 21]. In addition, the *TERTp* mutations are well established to increase *TERT* expression and its activity, with a tissue-specific distribution in several cancers. Killela et al. [32] reported that the frequency of *TERTp* mutations is different in various cancer types: constantly self-renewing cancers, including breast cancer, harbor few of these telomere-maintaining mutations, whereas cancers arising from seldom self-renewing cells such as gliomas often harbor these mutations. Therefore, the role of rs2853669 likely depends on the frequency of *TERTp* mutations in different cancers, thereby demonstrating a tissue-specific effect on cancer risk. Several other cancers such as lung cancer and glioblastoma were also analyzed by stratification; however, each type of cancer only contained one or two studies, which unfortunately provided insufficient study numbers for a meta-analysis. Thus in the future, more large-scale studies are warranted to further elucidate the role of rs2853669 in different cancers.

Several recent studies [8, 11, 15–18, 21, 22] reported inconsistent results about the modifying effect of rs2853669 on *TERTp* mutations in cancer survival, so we also conducted a meta-analysis to address this issue. Results revealed that the poor prognosis contributed by *TERTp* mutations occurs only in cancer patients carrying the rs2853669 TT genotype; whereas in patients carrying the rs2853669 C allele (i.e., the TC or CC genotype), *TERTp* mutations showed no effect on the OS or DFS. Subgroup analyses based on cancer type was consistent with the overall analysis result. The rs2853669 polymorphism is located within a preexisting Ets2 transcription factor binding site in the promoter region of the *TERT* gene [8]; therefore, the C allele variant may impair the Ets2 binding site and then prevent c-Myc from binding to the *TERT* E-box. This factor decreases *TERT* expression and lowers telomerase activity, thereby blunting the detrimental effect of *TERTp* mutations [21, 33, 34].

Our study included 143,107 subjects and 2,234 cases for evaluating the effect of rs2853669 alone on cancer risk and prognosis, respectively, and included 1,775 cases for further evaluating the modifying effect of rs2853669 on *TERTp* mutations in the survival of cancer patients. A large sample size and systematic assessment could provide the exact role of rs2853669 in cancer onset and development. However, several limitations need to be addressed. First, many types of cancer were included in our meta-analysis, but the majority only contained one or two studies; therefore, the subgroup analyses were not fully implemented for each type of cancer. Second, one-way sensitivity analysis showed that the significant result obtained for the modifying effect of rs2853669 was not stable. This finding should therefore be treated with caution. Third, our pooled results may be biased by residual or unmeasured confounders in the original studies. Thus it also should be cautious about the prognostic role of rs2553669 in cancer, because age, TNM stages, and treatment were not fully adjusted in original studies.

In conclusion, our meta-analysis demonstrated that rs2853669 alone does not increase or decrease the overall risk and prognosis of cancer. Moreover, the prognostic value of *TERTp* mutations significantly depends on the rs2853669 status: cancer patients with *TERTp* mutations who carry the TT genotype have a poor survival. Our findings reveal the potential role of rs2853669 in carcinogenesis, and provide evidence for the clinical utility of the combination of rs2853669 and *TERTp* mutations as biomarkers of cancer prognosis. However, more cohort studies with refined designs are required to further validate our results.

## MATERIALS AND METHODS

### Search strategy and eligibility criteria

We searched literatures through PubMed, Web of Science, and EMBASE up to July 2016. The search items included “rs2853669,” “*TERT* or *hTERT*,” “polymorphism or variant,” and “cancer or tumor or carcinoma or neoplasm or malignancy”. Our report was conducted in accordance with the guidelines of Preferred Reporting Items for Systematic Reviews and Meta-Analyses [35]. Eligible studies were included if they met the following criteria: (1) studies had a case-control or cohort design; (2) studies focusing on the association of *TERT*-rs2853669 with cancer risk or prognosis were published in English; and (3) studies reported the ORs or HRs with the corresponding 95%CIs, or studies provided sufficient information to calculate the ORs or HRs. If studies had overlapping case series, we used the study with the latest or largest sample size.

### Data extraction and quality assessment

Two authors independently extracted data from each eligible study, and discrepancies were resolved through discussion and consensus. Extracted items included the first author, publication year, country, cancer type, sex, number of subjects and so on. We resolved any discrepancy by group discussion. For cancer risk, we assessed the quality of each study by using the quality assessment criteria (Supplementary Table 1) with scores ranging from “0” (i.e., worst) to “15” (i.e., best); scores ≤9 indicated a low quality and scores >9 indicated a high quality [36]. For cancer prognosis, we did not assign a quality scale for each study because there is no such score assessment having a general consensus in a prognostic meta-analysis for observational studies; we instead carried out the subgroup and sensitivity analyses to evaluate the potential effects of rs2853669, according to the previous meta-analyses [37, 38].

### Statistical analysis

We applied a random-effects model to pool data [39]. Adjusted effect measures were chosen preferentially, if available. When an individual study did not provide effect measures, we calculated the OR and 95% CI by the allele frequencies, and extrapolated the HR and 95%CI by the methods of Parmar [40] and Tierney [41]. Hardy-Weinberg equilibrium was re-examined by χ^2^ test in controls. The heterogeneity was assessed by the Cochran's Q test and quantified by *I^2^* statistics, in which *P* < 0.10 or *I^2^* > 50% indicated significant heterogeneity. Meta-regression analysis was applied to investigate potential sources of heterogeneity. We also performed one-way sensitivity analyses to evaluate the stability of the pooled results. In addition, we performed the Begg's test [42] and Egger's test [43] to examine publication bias. All aforementioned statistical analyses were performed via Stata 12.1 software (StataCorp, College Station, TX, USA), using a two-sided *P* ≤ 0.05 as the significance level, unless otherwise specified.

## SUPPLEMENTARY MATERIALS FIGURES AND TABLE



## Abbreviations

CI: confidence interval
DFS: disease-free survival
HR: hazard ratio
OR: odds ratio
OS: overall survival
SNP: single nucleotide polymorphism
TERT: telomerase reverse transcriptase.
