# Supplementary material for: Association between rs2853669 in TERT gene and the risk and prognosis of human cancer: a systematic review and meta-analysis
Source: Oncotarget. 2017 Feb 7;8(31):50864–72. doi: 10.18632/oncotarget.15140 (PMC5584211; doi:10.18632/oncotarget.15140)
Supplement: Supplementary file 1 [file oncotarget-08-50864-s001.pdf]

Association between rs2853669 in *TERT* gene and the risk and prognosis of human cancer: a systematic review and meta-analysis

SUPPLEMENTARY MATERIALS

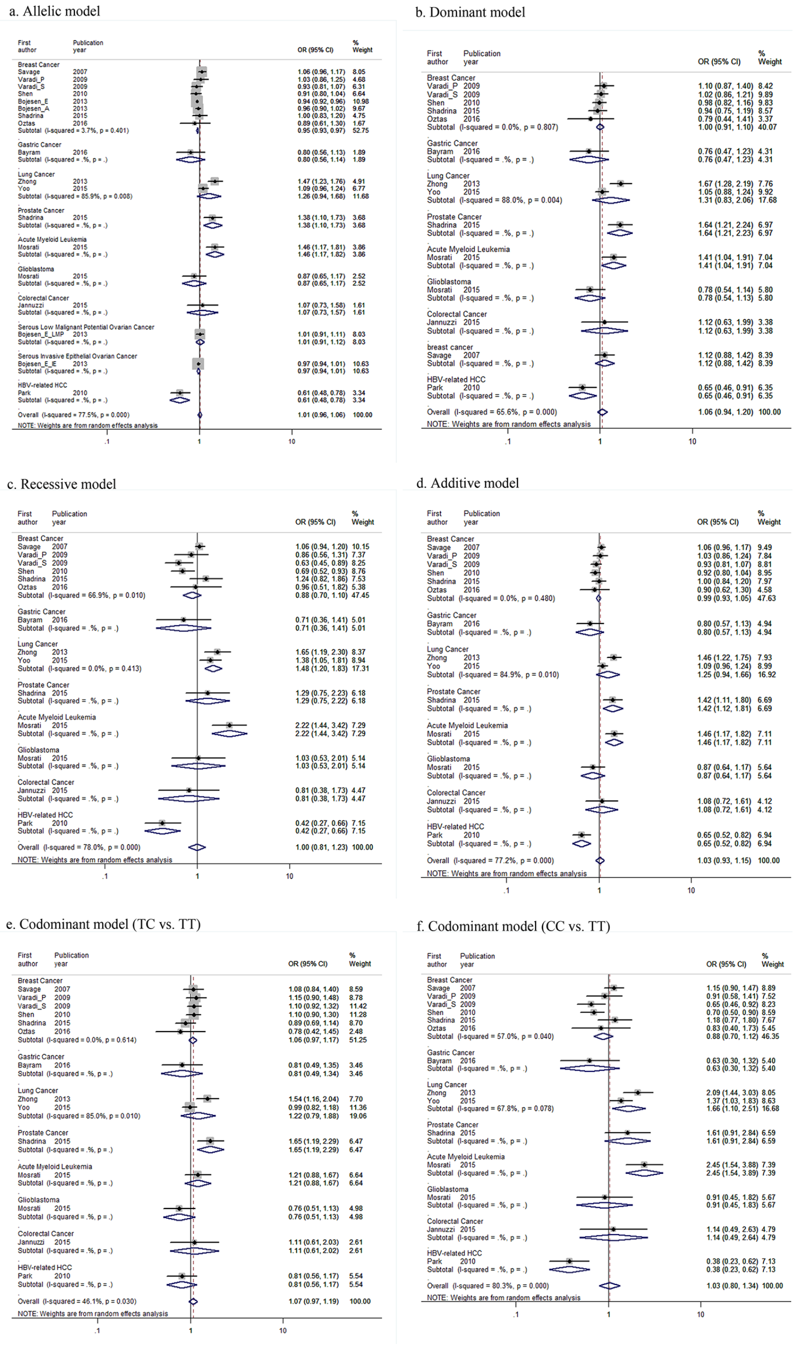

Supplementary Figure 1: Forest plots of the association between rs2853669 and overall cancer risk in the **a.** allelic, **b.** dominant, **c.** recessive, **d.** additive, **e.** TC vs. TT codominant and **f.** CC vs. TT codominant model. Varadi_P, Polish population in the study of Varadi, et al.; Varadi_S, Swedish population in the study of Varadi, et al.; Bojesen_E, Europeans in the study of Bojesen, et al.; Bojesen_A, Asians and African-Americans in the study of Bojesen, et al.; Bojesen_E_LMP, low malignant potential ovarian cancer in Europeans in the study of Bojesen, et al.; Bojesen_E_IE, invasive epithelial ovarian cancer in Europeans in the study of Bojesen, et al.; HCC, hepatocarcinoma.

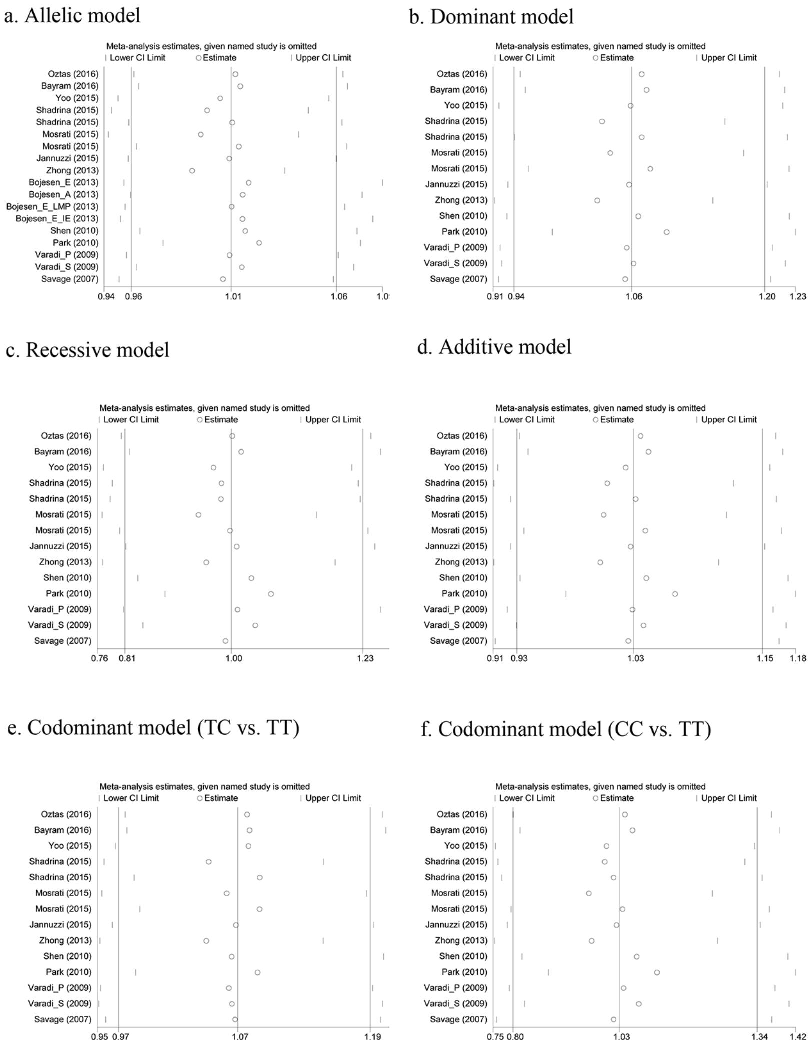

Supplementary Figure 2: Sensitivities analyses plots of the association between rs2853669 and overall cancer risk in the **a.** allelic, **b.** dominant, **c.** recessive, **d.** additive, **e.** TC vs. TT codominant and **f.** CC vs. TT codominant model. Varadi P, Polish population in the study of Varadi, et al.; Varadi_S, Swedish population in the study of Varadi, et al.; Bojesen_E, Europeans in the study of Bojesen, et al.; Bojesen_A, Asians and African-Americans in the study of Bojesen, et al.; Bojesen_E_LMP, low malignant potential ovarian cancer in Europeans in the study of Bojesen, et al.; Bojesen_E_IE, invasive epithelial ovarian cancer in Europeans in the study of Bojesen, et al.

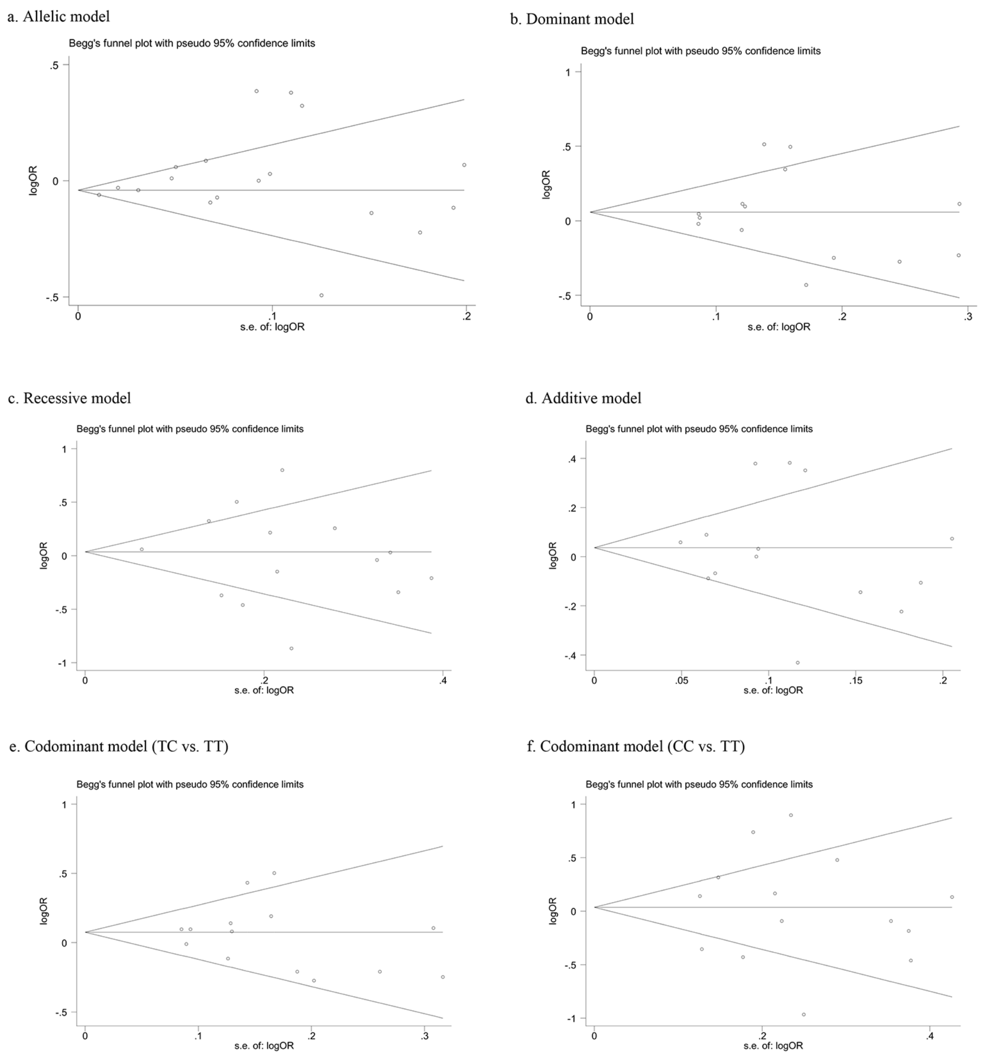

Supplementary Figure 3: Funnel plots of the association between rs2853669 and overall cancer risk in the **a.** allelic, **b.** dominant, **c.** recessive, **d.** additive, **e.** TC vs. TT codominant and **f.** CC vs. TT codominant model.

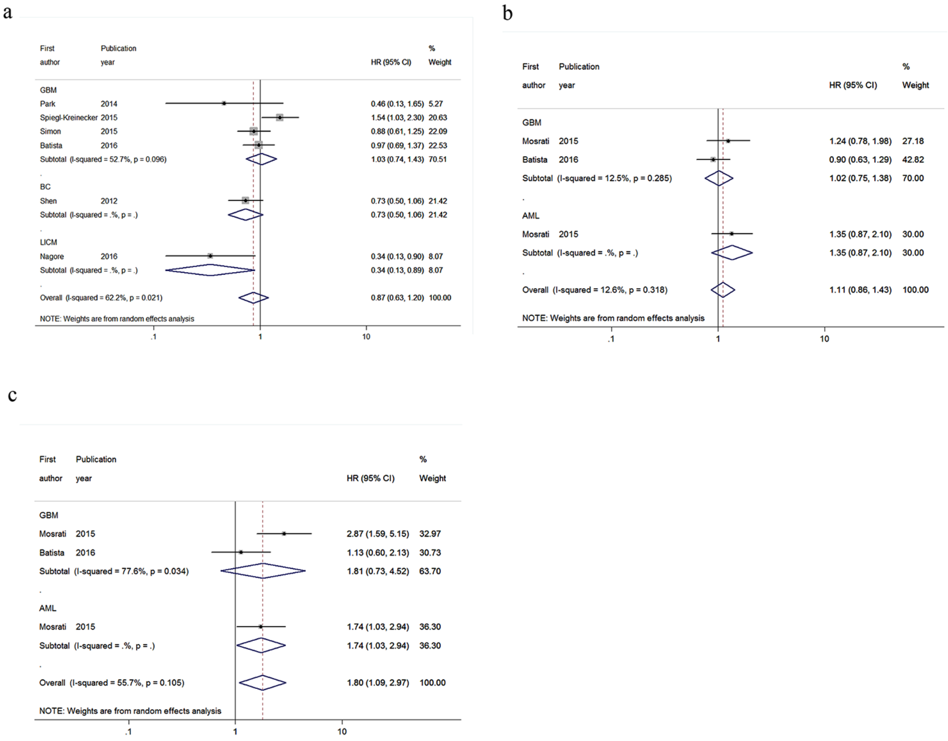

Supplementary Figure 4: Forest plots of the association between rs2853669 and overall survival of cancer in the **a.** dominant, **b.** TC vs. TT codominant and **c.** CC vs. TT codominant model. GBM, glioblastoma; BC, breast cancer; LICM, localized invasive cutaneous melanoma; AML, acute myeloid leukemia.

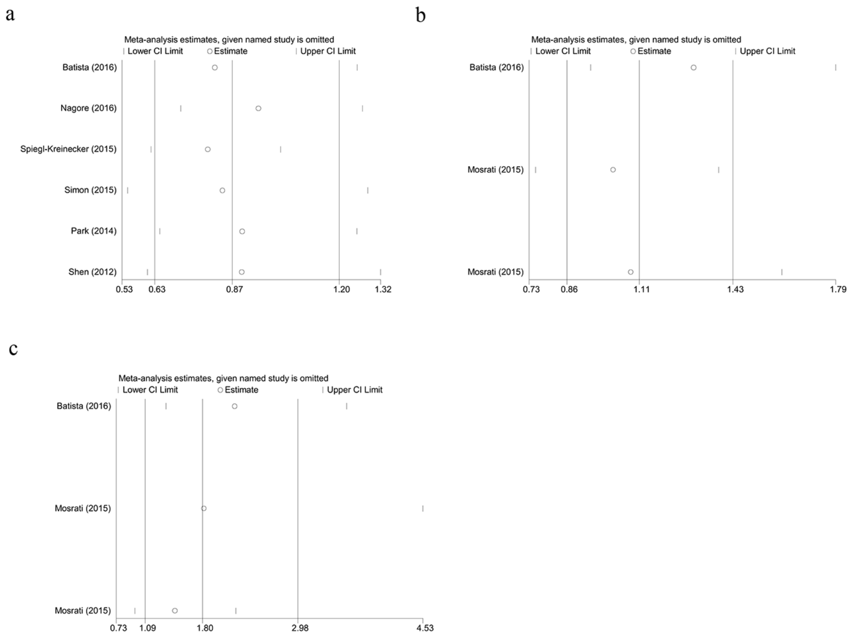

Supplementary Figure 5: Sensitivities analyses plots of the association between rs2853669 and overall survival of cancer in the **a.** dominant, **b.** TC vs. TT codominant and **c.** CC vs. TT codominant model.

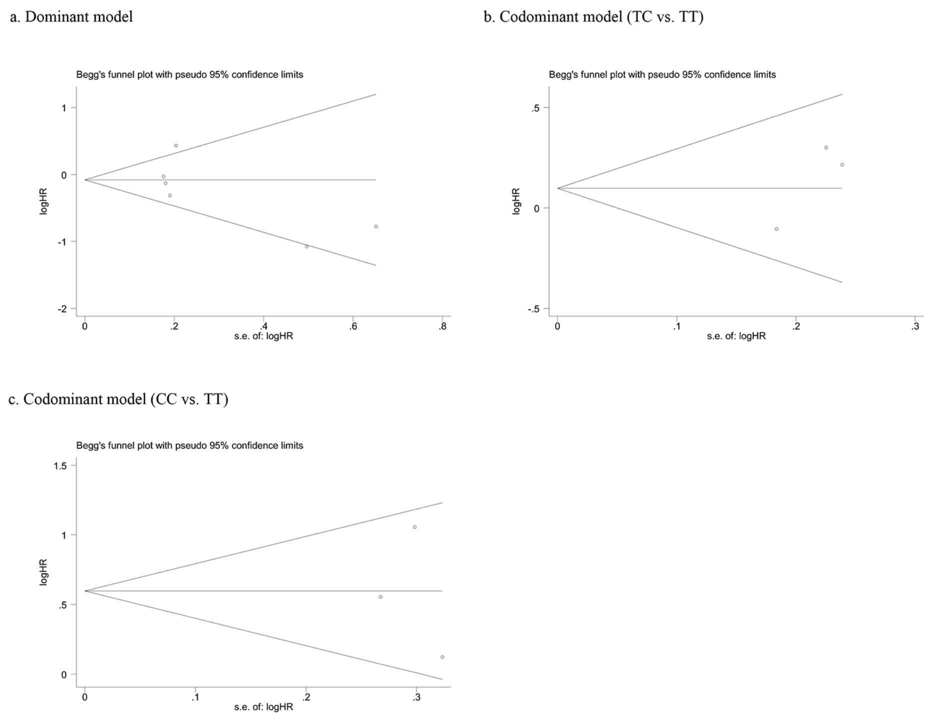

Supplementary Figure 6: Funnel plots of the association between rs2853669 and overall survival of cancer in the **a.** dominant, **b.** TC vs. TT codominant and **c.** CC vs. TT codominant model.

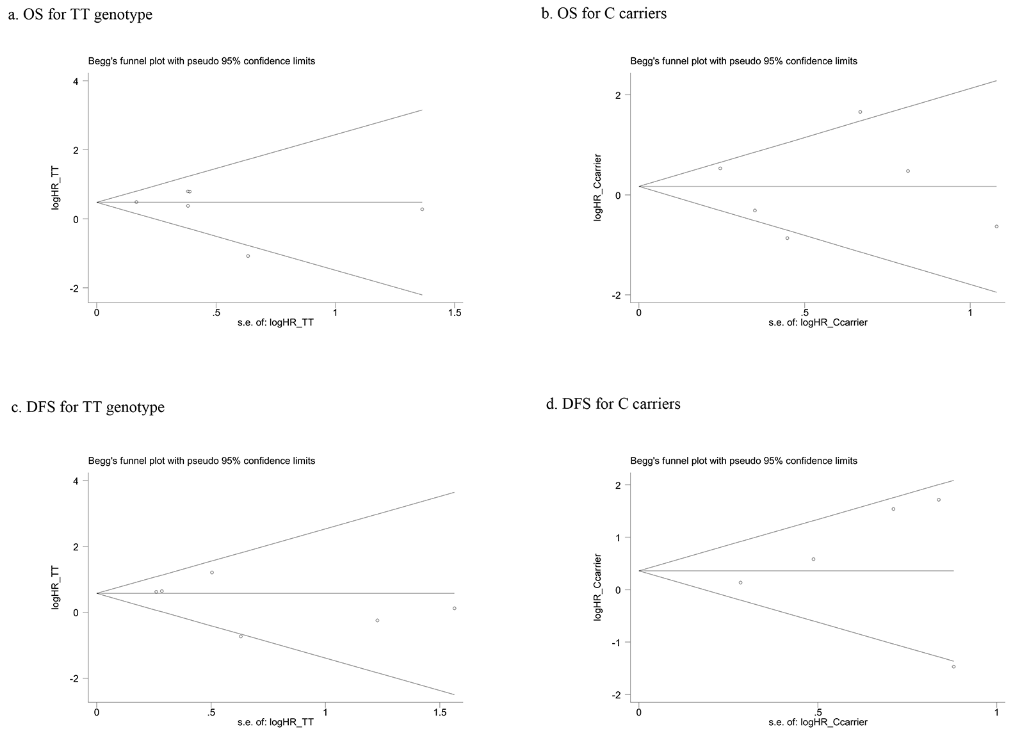

Supplementary Figure 7: Funnel plots of the modifying effect of rs2853669 on *TERTp* mutations for **a.** overall survival and **c.** disease-free survival in cancer patients carrying TT genotype, and for **b.** overall survival and **d.** disease-free survival in cancer patients carrying TC or CC genotype. OS, overall survival; DFS, disease-free survival.

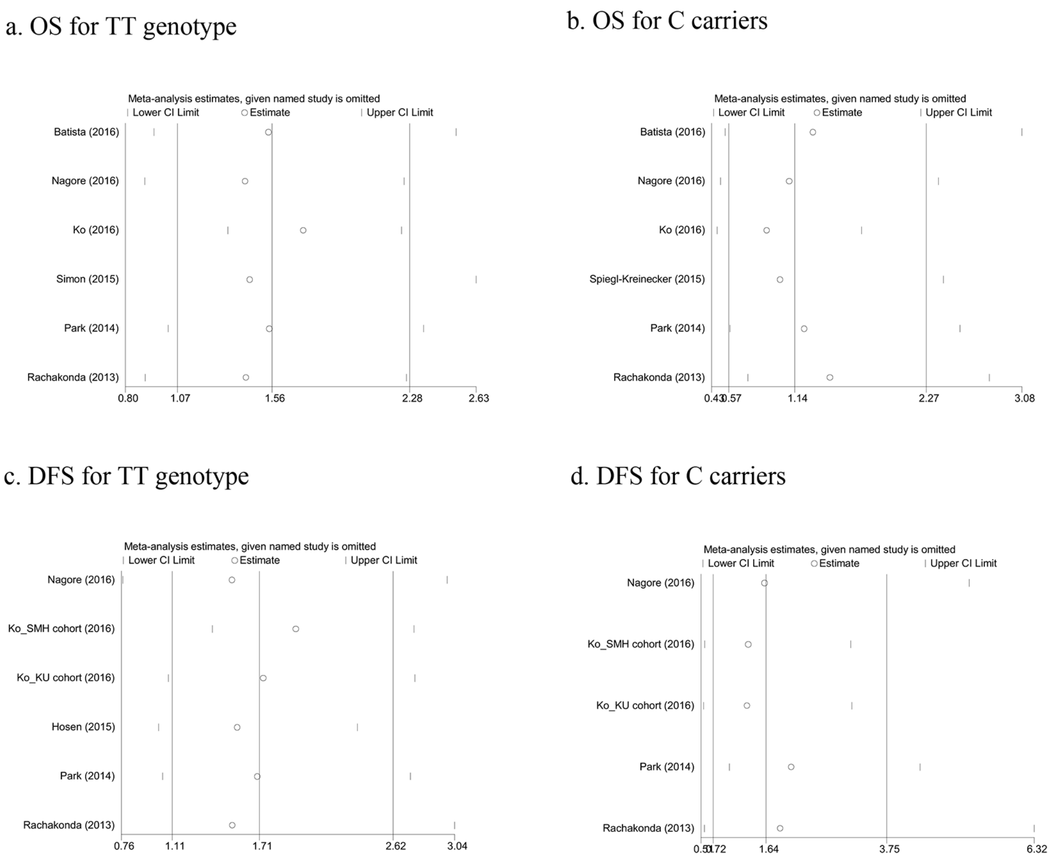

Supplementary Figure 8: Sensitivities analyses plots of the modifying effect of rs2853669 on *TERTp* mutations for **a.** overall survival and **c.** disease-free survival in cancer patients carrying TT genotype, and for **b.** overall survival and **d.** disease-free survival in cancer patients carrying TC or CC genotype. OS, overall survival; DFS, disease-free survival.

Supplementary Table 1: Criteria of quality assessment score

Criteria	Scores
**Representativeness of case**	
Selected from cohort or cancer registry	3
Selected from hospital or clinic centers	2
Selected from self-report data	1
No method of selection described	0
**Representativeness of control**	
Population-based	3
Blood donors	2
Hospital-based	1
Not described	0
**Case ascertainment**	
Histopathologic confirmation	2
Patient medical record	1
Not described	0
**Control selection**	
Controls matched with cases by age and sex	2
Controls matched with cases only by age or by sex	1
Not matched or not descried	0
**Adjustment of odds ratio (OR)**	
OR adjusted by confounders	1
Not described	0
**Hardy-Weinberg equilibrium (HWE) in controls**	
HWE	1
Deviation from HWE or not mentioned	0
**Total sample size**	
> 1000	3
501 - 1000	2
201 - 500	1
≤ 200	0
